# Unusual Manifestation of a Non-inflamed Appendiceal Fistula as a Necrotizing Soft Tissue Infection in the Setting of a Gigantic Ventral Hernia

**DOI:** 10.7759/cureus.28578

**Published:** 2022-08-30

**Authors:** Elena J Danielson, Dustin J Nowotny, Mentor Ahmeti

**Affiliations:** 1 General Surgery, University of North Dakota School of Medicine and Health Sciences, Grand Forks, USA; 2 Trauma and Acute Care Surgery, Sanford Health, Fargo, USA

**Keywords:** appendico-cutaneous fistula, colocutaneous fistula, appendiceal fistula, necrotizing soft tissue infection, abdominal wall necrotizing fasciitis, abdomen ventral hernia

## Abstract

Necrotizing soft tissue infection (NSTI) is a rapidly progressive infection of the soft tissues that necessitates early identification and emergent aggressive surgical debridement due to its high mortality. NSTI most often results from the introduction of microbes through breaks in the skin. Unique sources, like appendiceal fistulae, can be etiologies of abdominal wall NSTIs. We present the case of a 46-year-old female with a past medical history of poorly controlled type II diabetes mellitus and ventral hernia who presented in septic shock with a necrotic wound in her abdominal wall. The wound was overlying a large ventral hernia and was consistent with NSTI. She was treated urgently with fluid resuscitation, antibiotic therapy, and surgical debridement of the wound. On repeat exploration, an appendiceal fistula was found protruding from the hernial sac. Open appendectomy and primary repair of the ventral hernia were performed. Principles of immediate intervention and repeat surgical debridement allowed control of the septic insult and definitive source control upon identification of an appendiceal fistula.

## Introduction

A necrotizing soft tissue infection (NSTI) is an infection involving the skin, subcutaneous tissue, fascia, and/or muscle that is characterized by rapidly progressive tissue destruction, systemic toxicity, and high mortality [1-2]. Presentation of NSTIs can vary from erythema and pain that initially may resemble uncomplicated cellulitis, to necrosis and septic shock in advanced disease states. Early, aggressive surgical intervention is necessary for managing NSTIs, and early, frequent repeat debridement is indicated to ensure all infectious and necrotic tissue has been removed.

NSTIs typically develop after the introduction of microbes through breaks in the skin. These infections most frequently affect the extremities, however, nearly any site of the body can be involved. NSTIs of the abdominal wall may be seen as a complication following abdominal surgeries, as in Meleney’s synergistic gangrene. Uncommonly, they may be due to enterocutaneous fistula formation after bowel surgery or in other intra-abdominal inflammatory processes. Appendiceal fistulae are the most uncommon type of enteric fistula [3-4]. Specifically, appendico-cutaneous fistulae, though rare, have previously been described in the literature [4]. The most common cause of appendiceal fistulae is acute appendicitis [4]. Although acute appendicitis is typically due to an obstructing fecalith or tumor in adults, it may also occur as a result of incarceration of the appendix within an abdominal wall or inguinal hernia.

## Case presentation

**Figure 1:**
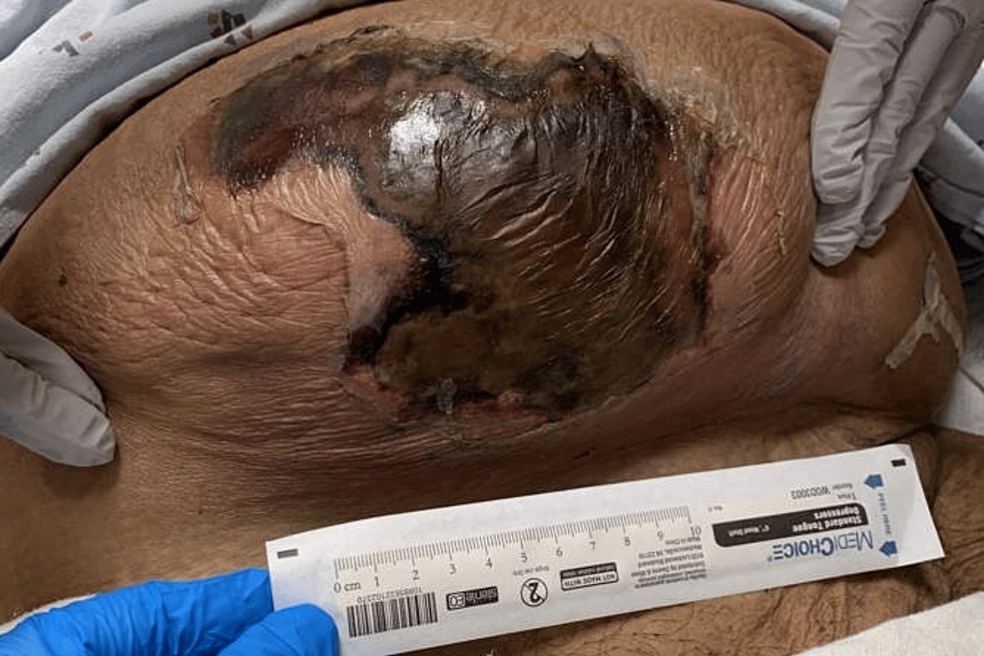
Lower abdominal wall wound at the time of initial presentation

**Figure 2:**
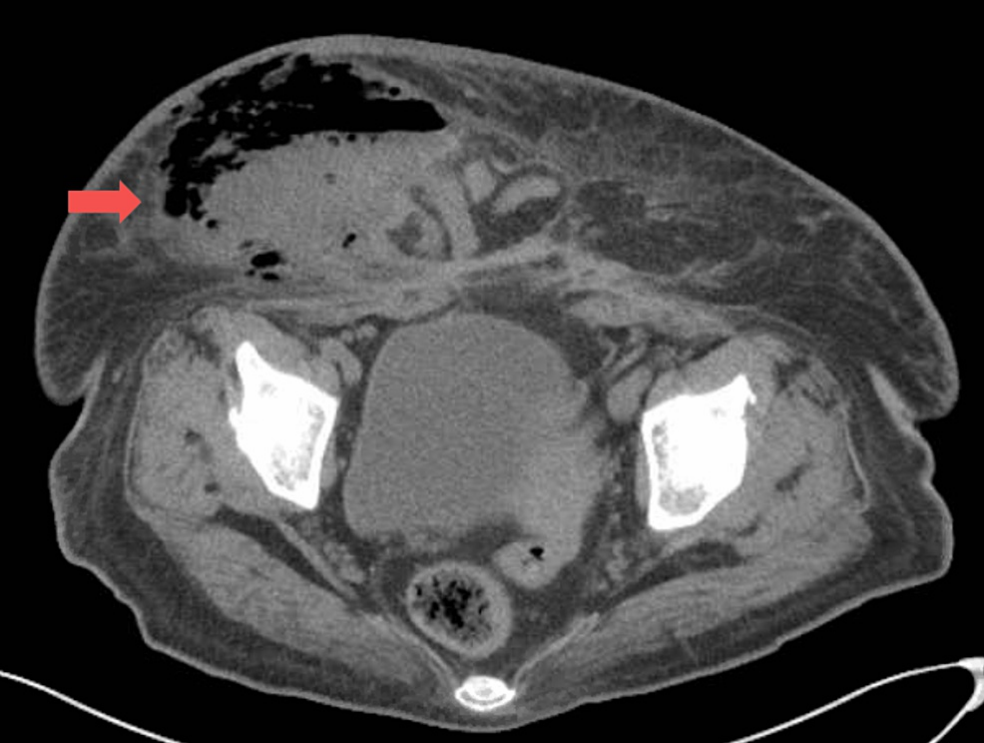
CT image demonstrating a large ventral hernia with an overlying abscess and prominent, associated soft tissue gas (arrow)

**Figure 3:**
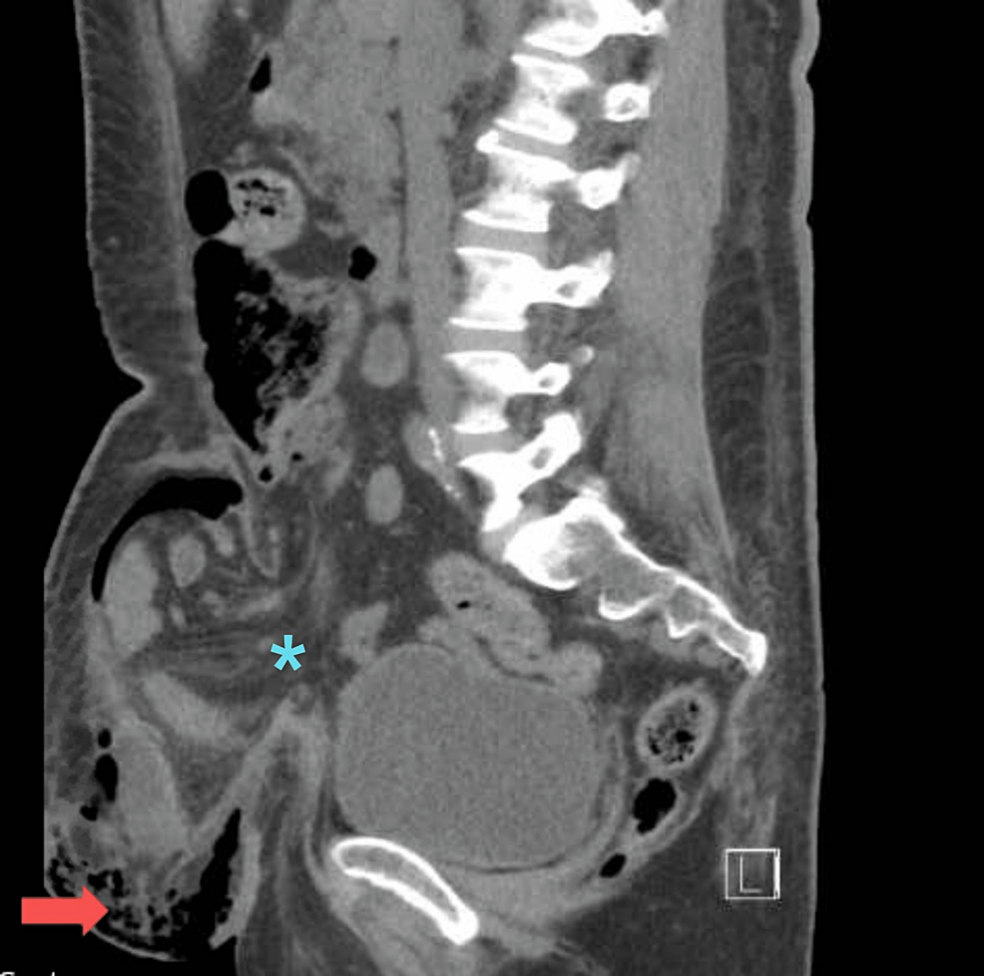
CT image demonstrating a large ventral hernia (asterisk) with overlying abscess and prominent associated soft tissue gas (arrow)

A 46-year-old female with a past medical history of poorly controlled type II diabetes mellitus, obesity, current tobacco use, prior laparoscopic cholecystectomy, and cesarean section, with a large incisional ventral hernia presented to her local hospital in septic shock with a necrotic wound on her torso. She started having abdominal pain over the right lower abdomen one week prior to her presentation. She noticed a wound a few days later, which became black, and started expressing purulent, malodorous drainage. She also started having fevers and malaise. The patient was obese and had a large necrotic wound over the right lower abdominal wall (Figure 1). Laboratory workup revealed leukocytosis (white blood cell count 27.1 x 10^9^/L), hyponatremia (sodium 128 mmol/L), acute kidney injury (creatinine 159.12 μmol/L), and hyperglycemia (glucose 29.53 mmol/L). A computed tomography (CT) scan of the abdomen and pelvis revealed a large ventral hernia containing multiple loops of normal-appearing small bowel. There was a 13 cm wide abscess with associated prominent soft tissue gas extending to the hernial cavity and the right abdominal wall (Figures 2-3).

The patient was started on intravenous piperacillin-tazobactam, vancomycin, and clindamycin for broad-spectrum antibiotic coverage. She was taken emergently to the operating room (OR) for debridement. Necrotic tissues were sharply debrided back to healthy tissue. A portion of the hernial sac was opened revealing no purulent fluid within the hernia nor intra-abdominally. Bowel appeared adhered together but viable and without signs of perforation. Due to continued hemodynamic instability, the patient was left intubated with plans to return to OR once stable.

Within several hours, there was increased erythema and warmth of the abdomen surrounding the wound, necessitating an emergent return to the OR. Re-exploration was performed with extensive debridement of necrotic tissues, including fascia. During this second debridement, the peritoneal cavity was explored through the prior opening of the hernial sac. The exposed bowel was noted to have significant adhesions but once again appeared viable. The abdomen was left open, and the wound was packed with wet-dry dressings. The patient returned to the intensive care unit (ICU) in critical condition. She remained on IV antibiotic therapy, and her clinical status started to show improvement.

Once hemodynamically stable, she was taken back to the OR for the third time for re-exploration. Initial examination of the wound showed a small, prolapsed piece of mucosal tissue protruding through the remaining hernial sac. The hernial sac was fully opened, and the abdominal cavity was entered through the sac. Lysis of adhesions was performed to release the small bowel from the abdominal wall to allow for close inspection of the bowel. Following identification of the ileum and cecum, the appendix was identified and found to have a defect with exposed mucosa through the hernial sac. The exposed mucosa was identified as the prolapsed piece of tissue that was initially seen protruding from the hernia, consistent with an appendiceal fistula that contributed to her initial necrotizing soft tissue infection. An open appendectomy was performed. The examined bowel was found to be viable. After resection of the hernial sac, the transverse fascial defect measured eight cm long by 15 cm wide. In the setting of class III, i.e. contaminated wound, the defect was closed primarily. Minimal additional debridement of the soft tissues was performed. The abdominal wound was then packed with gauze and allowed to heal by secondary intention. After the third procedure, the patient recovered rapidly and was discharged home four days later.

Cultures obtained from the abdominal wound during the initial procedure grew mixed aerobic and anaerobic flora consistent with intestinal flora. Specifically, aerobic culture grew many *Streptococcus anginosus*, few *Klebsiella pneumoniae,* and few *Escherichia coli*. Pathologic examination of the soft tissue samples confirmed necrotizing fasciitis. Pathological examination of the appendix revealed a benign appendix with acute peri-appendicitis and serositis but without transmural appendicitis. The presence of an appendiceal fistula in the tip of the appendix was confirmed.

At the six-week follow-up appointment, the wound appeared to be healing well without signs of infection.

## Discussion

Incarceration of the appendix within hernias is unusual but well-described in the literature, dating back to 1735 when Claudius Amyand described a case of an inguinal hernia with an incarcerated appendix [5]. Even rarer than incarceration of the appendix in the inguinal hernia is incarceration within a ventral hernia. This has only been described in a few case reports [6]. Incarceration of the appendix can lead to complications including strangulation, acute appendicitis, perforation, and, very rarely, fistulization.

Appendiceal fistulae are an uncommon type of enteric fistulae. They are defined as the primary perforation of the appendix into an adjacent hollow viscus or skin [4]. Appendico-cutaneous fistulae are rare but have been described as complications of appendiceal abscess drainage, incomplete appendectomy, or as spontaneous complications of acute appendicitis. Acute appendicitis, particularly when associated with a fecalith or an obstructing neoplasm, is the most commonly identified source of appendiceal fistulae in a review by McDonald et al. [4].

NSTI due to appendicitis with perforation is an infrequent but severe complication [7-8]. NSTI due to an appendico-cutaneous fistula is extremely rare but has been previously described in a few case reports, including two cases with incarcerated hernias acting as the site of fistulization [9-10].

The uniqueness of the present case lies in the fact that a large ventral hernia with multiple bowel loops and the appendix as its contents had formed a fistula with the anterior abdominal wall and necrotized the skin, which was not identified by imaging or by initial surgical exploration. There were signs of intra-abdominal inflammation with adhesions between loops of the bowel, but the bowel did not appear acutely distressed. Furthermore, there was no apparent inciting acute appendicitis or other inflammatory processes such as an abscess within the abdomen that led to fistulization.

NSTIs are aggressive subcutaneous infections with high mortality. The cornerstone of treatment is early and aggressive surgical debridement [1-2,11]. After initial stabilization, the underlying source can be identified. Although uncommon, an intra-abdominal source of infection needs to be considered in the case of abdominal wall NSTI. After the repeated debridement of necrotic tissues in this case, definitive source control was obtained with the successful removal of the fistula through an open appendectomy. Closure of the abdominal wall was achieved with primary repair of the ventral hernia during the same operation.

## Conclusions

We present a unique case of an appendico-cutaneous fistula within an incarcerated ventral hernia presenting as an NSTI of the abdominal wall. Although uncommon, it is important to consider intra-abdominal pathology as a source of NSTI in the abdominal wall. An appendiceal fistula is an unusual cause of an NSTI. However, successful treatment still depends on the principle of source control with early, aggressive surgical debridement and, in this case, removal of the fistula via appendectomy.
